# Interspecific Signaling Between the Parasitic Plant and the Host Plants Regulate Xylem Vessel Cell Differentiation in Haustoria of *Cuscuta campestris*

**DOI:** 10.3389/fpls.2020.00193

**Published:** 2020-03-13

**Authors:** Yuki Kaga, Ryusuke Yokoyama, Ryosuke Sano, Misato Ohtani, Taku Demura, Takeshi Kuroha, Naoki Shinohara, Kazuhiko Nishitani

**Affiliations:** ^1^Graduate School of Life Sciences, Tohoku University, Sendai, Japan; ^2^Graduate School of Science and Technology, Nara Institute of Science and Technology, Ikoma, Japan; ^3^Graduate School of Frontier Sciences, The University of Tokyo, Kashiwa, Japan; ^4^Faculty of Science, Kanagawa University, Hiratsuka, Kanagawa, Japan

**Keywords:** stem parasitic plant, plant-plant interspecific interaction, haustorium development, xylem vessel differentiation, transcriptome analysis

## Abstract

The genus *Cuscuta* is stem parasitic angiosperms that parasitize a wide range of vascular plants via de novo formation of a distinctive parasitic organ called a haustorium. In the developing haustorium, meristematic cells, which are initiated from the stem cortical tissue, differentiate into haustorial parenchyma cells, which elongate, penetrate into the host tissues, and finally connect with the host vasculature. This interspecific vasculature connection allows the parasite to uptake water and nutrients from the host plant. Although histological aspects of haustorium development have been studied extensively, the molecular mechanisms underlying vasculature development and the interspecific connection with the host vasculature remain largely unknown. To gain insights into the interspecific cell-to-cell interactions involved in haustorium development, we established an *in vitro* haustorium induction system for *Cuscuta campestris* using *Arabidopsis thaliana* rosette leaves as the host plant tissue. The *in vitro* induction system was used to show that interaction with host tissue was required for the differentiation of parasite haustorial cells into xylem vessel cells. To further characterize the molecular events occurring during host-dependent xylem vessel cell differentiation in *C. campestris*, we performed a transcriptome analysis using samples from the *in vitro* induction system. The results showed that orthologs of genes involved in development and proliferation of vascular stem cells were up-regulated even in the absence of host tissue, whereas orthologs of genes required for xylem vessel cell differentiation were up-regulated only after some haustorial cells had elongated and contacted the host xylem. Consistent results were obtained by another transcriptome analysis of the haustorium development in *C. campestris* undergoing parasitization of an intact host plant. These findings suggest the involvement of host-derived signals in the regulation of non-autonomous xylem vessel differentiation and suggest that its connection to the host xylem during the haustorium development activates a set of key genes for differentiation into xylem vessel cells.

## Introduction

Although land plants originated as autotrophic organisms, some angiosperms have evolved parasitism. Parasitic angiosperms have acquired the ability to absorb water and nutrients from host plants through an invasive organ called a haustorium (Westwood et al., 2010). Parasitic angiosperms are categorized by the degree of their dependency on the host plant for nutrients. Hemiparasitic angiosperms have photosynthetic capacity and rely only partly on the host plant, while holoparasitic angiosperms lack this capacity and cannot survive without parasitizing the host plant (Heide-Jørgensen, 2008). The genus *Cuscuta* or dodder plants, which are classified in the family Convolvulaceae, lack roots and true leaves, and are considered to be holoparasitic angiosperms (Dawson et al., 1994).

After a *Cuscuta* stem coils around the stem of a host plant, the cortical tissue on the concave side of the *Cuscuta* stem, in contact with stem surface of the host plant, begins to proliferate and expand to form a haustorial meristem (Dawson et al., 1994). Two types of cell differentiate within the meristem: tip cells (apical side) and file cells (proximal side) (Hong et al., 2011). As haustorium development proceeds, tip cells and file cells grow into search hyphae and axial cells, respectively (Hong et al., 2011), and the haustorium begins to penetrate into the host epidermal tissues. Penetration is facilitated by enzymatic cell-wall degradation and driven by the force generated by cell division and cell elongation in the axial cell region (Nagar et al., 1984; Dawson et al., 1994). After the penetration event, search hyphae begin to elongate extensively by tip growth in the host tissue (Dawson et al., 1994), and intrude into the host xylem, where they differentiate into xylem vessel cells (also termed xylem hyphae) (Hong et al., 2011). Connections between host and parasite xylems have also been observed in mature haustoria (Birschwilks et al., 2007). However, it remains unclear how xylem differentiation is regulated and how the xylem connection is established between the host plant and the parasitic plant.

During vasculature development in angiosperms, xylem vessel cell formation is initiated by differentiation of vascular stem cells under the regulation of MONOPTEROS (MP), which belongs to a family of auxin-responsive factors (ARFs). MP directly activates the expression of *ARABIDOPSIS THALIANA HOMEOBOX8 (ATHB8)* (Schlereth et al., 2010), which encodes a transcription factor that induces the expression of the *PIN-FORMED 1* (*PIN1*) gene and activates the development of pre-procambial cells (Scarpella et al., 2006). Additionally, MP directly activates the expression of *TARGET OF MONOPTEROS* and *TMO5-LIKE1* (*TMO5* and *T5L1*) (Scarpella et al., 2006). A heterodimeric complex of TMO5/T5L1 and LONESOME HIGHWAY (LHW) promotes cytokinin biosynthesis in cells surrounding xylem precursor cells by triggering the transcription of *LONELY GUY3* and *LONELY GUY4* (*LOG3* and *LOG4*), resulting in the regulation of cell division and patterning in vascular tissues (De Rybel et al., 2013; Ohashi-Ito et al., 2014). Phytohormones including auxins and cytokinins are involved in xylem vessel formation. Brassinosteroids also play a role in xylem vessel formation by promoting the transcription of HD-ZIP III transcription factor family genes, which are involved in establishing vascular patterning and determining cell fate (Ohashi-Ito et al., 2002; Fukuda, 2004). After the determination of cell fate in vascular tissue, the VASCULAR-RELATED NAC-DOMAIN (VND) family of transcription factors activates the expression of a set of genes required for xylem vessel cell differentiation (Kubo et al., 2005; Tan et al., 2018). The final process in xylem vessel differentiation is formation of patterned secondary cell walls (SCWs) and programmed cell death (PCD) (Fukuda, 2004).

Although the formation of xylem vessels in angiosperms is well understood, relatively little is known regarding haustorium and xylem development in parasitic plant genera such as *Cuscuta*. In an attempt to identify key genes responsible for the development of haustoria, transcriptome analyses have been performed that compared the expression profiles of different developmental stages of the *Cuscuta* haustoria. Genes involved in response to stimulus, transport activity, and cell wall functions exhibited high expression during haustorial development (Ranjan et al., 2014; Ikeue et al., 2015; Olsen et al., 2016). Despite these extensive studies, the molecular mechanisms regulating xylem differentiation during haustorium formation are still poorly understood.

Given that haustorium development is a cell-non-autonomous process that is influenced by interspecific cell-to-cell interactions, it is necessary to distinguish the contributions of the parasite and host to understand haustorium development. Accordingly, to investigate the effect of host factors on the development of the parasitic haustorium and its penetration into host tissues, an *in vitro* system was developed to enable separation of host and parasitic factors. This *in vitro* parasitization system was used to analyze the effect of host tissues on the transcriptional regulation of haustorium development in *C. campestris* by comparing expression in the absence and presence of host tissue. The results showed that elongation of search hyphae was initiated irrespective of host-derived biological factors but that host-derived factors were required for further differentiation of search hyphae in the haustorium and for final differentiation into xylem vessel cells and connection to the host vasculature to complete the parasitic linkage.

## Materials and Methods

### Plant Materials

Seeds of *Arabidopsis thaliana* (L.) Heynh. accession Col-0 were sown on mineral wool (Rockwool B.V., Grodan) moistened with MGRL liquid medium (Tsukaya et al., 1991) and grown under continuous white light (45 μmol m^−2^ s^−1^) in a growth chamber at 22°C (Nippon Medical and Chemical Instruments, Co., Ltd.). Seeds of *Cuscuta campestris* Yuncker were soaked in concentrated sulfuric acid for 25 min at 22°C, washed with distilled water at 22°C five times, and placed on a filter paper (No.5A 90 mm, Toyo Roshi Kaisha, Ltd.) immersed in tap water for germination under continuous white light (45 μmol m^−2^ s^−1^) in the growth chamber at 22°C.

### Induction of Parasitism

After germination, 5-day-old seedlings of *C. campestris* were placed in a position to attach to the inflorescence stems of 4–5-week-old *A. thaliana* plants. Parasitism was induced under blue light (wavelength peak = 444 nm, 7 μmol m^−2^ s^−1^) in a growth chamber at 25°C for 2 days, after which plants were grown under continuous white light (45 μmol m^−2^ s^−1^) at 22°C.

Parasitism was also induced using excised lateral shoots from mature *C. campestris* plants. Lateral shoots (3 cm in length) with the apex attached were cut from mature *C. campestris* plants that had parasitized a host plant. Shoot segments were then attached to new inflorescence stems of 4–5-week-old *A. thaliana* using surgical tape (Micropore™ Surgical Tape, 3M Company) and parasitism induced under blue light at 25°C as for seedlings. The process of parasitism was recorded by time-lapse imaging (TLC200, Brinno). The time at which coiling of *C. campestris* around the host plant was complete was designated as 0 hours after coiling (hac).

### *In vitro* Haustorium Induction

**Figure 1:**
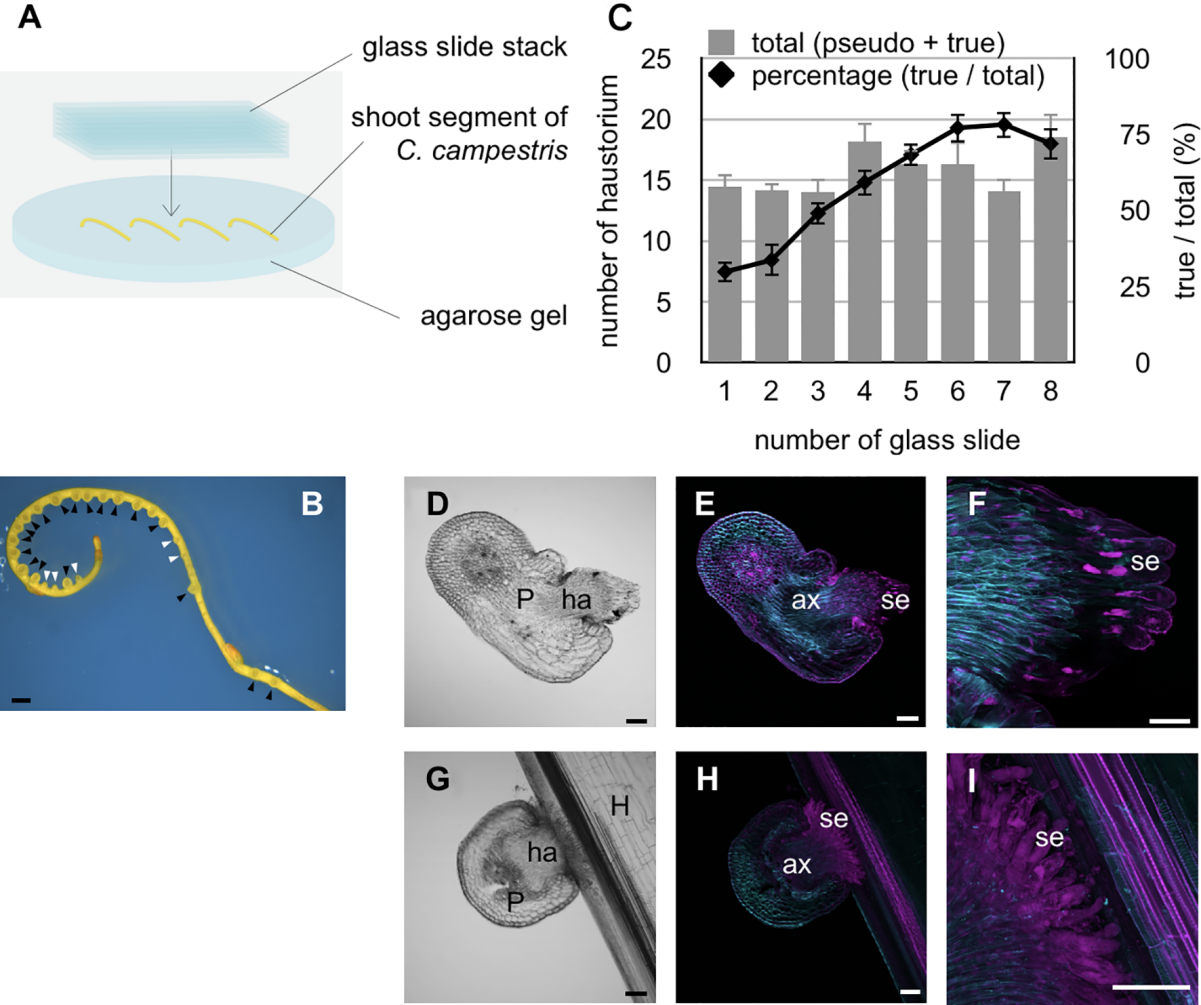
Physical pressure and blue light irradiation induce the protrusion of search hyphae during haustorium development. **(A)** Schematic of the *in vitro* haustorium induction procedure. **(B)** Formation of haustoria in an excised *C. campestris* lateral shoot that was pressed with a stack of glass slides under blue light (444 nm) irradiation at the dosage of 7 μmol m^−2^ s^−1^, and incubated at 25°C for 72 hours. Black arrowheads indicate true haustoria, while white arrowheads indicate pseudo haustoria. **(C)** Excised lateral shoots were placed on 3% agarose gel and pressure applied with a varying number of glass slides followed by incubation at 25°C for 72 hours. Total numbers of haustoria produced in each of the 3-cm long lateral shoot segments produced by the *in vitro* induction system, and percentage of true haustoria among the total haustoria, are shown as a function of the number of glass slides applied. Mean values from 11 biological replicates are shown with standard errors (SE) as vertical lines. **(D–F)** Images of a haustorium produced by the *in vitro* induction system at the stage of 72 hours after induction. **(D)** A bright-field image of a longitudinal section of the haustorium **(E, F)** Digital accumulation of fluorescence images of Z-serial optical sections of the same longitudinal section as for **(D)**. The section was double stained with Fluostain I and propidium iodide. **(F)** A high-magnification image of **(E)**. **(G–I)** Images of haustoria from *C. campestris* parasitizing an intact inflorescence stem of *A. thaliana* at the stage of 42 hours after coiling. **(G)** A bright-field image of a longitudinal section of the haustorium. **(H, I)** Digital accumulation fluorescence images of Z-serial optical sections of the same longitudinal section as for **(G)**, which was double stained with Fluostain I and propidium iodide. **(I)** High-magnification images of **(H)**. Scale bars: **(B)** 1 mm; **(D–I)** 100 μm. P, parasitic plant; ha, haustorium; ax, axial cell; se, search hypha; H, host plant.

**Figure 2:**
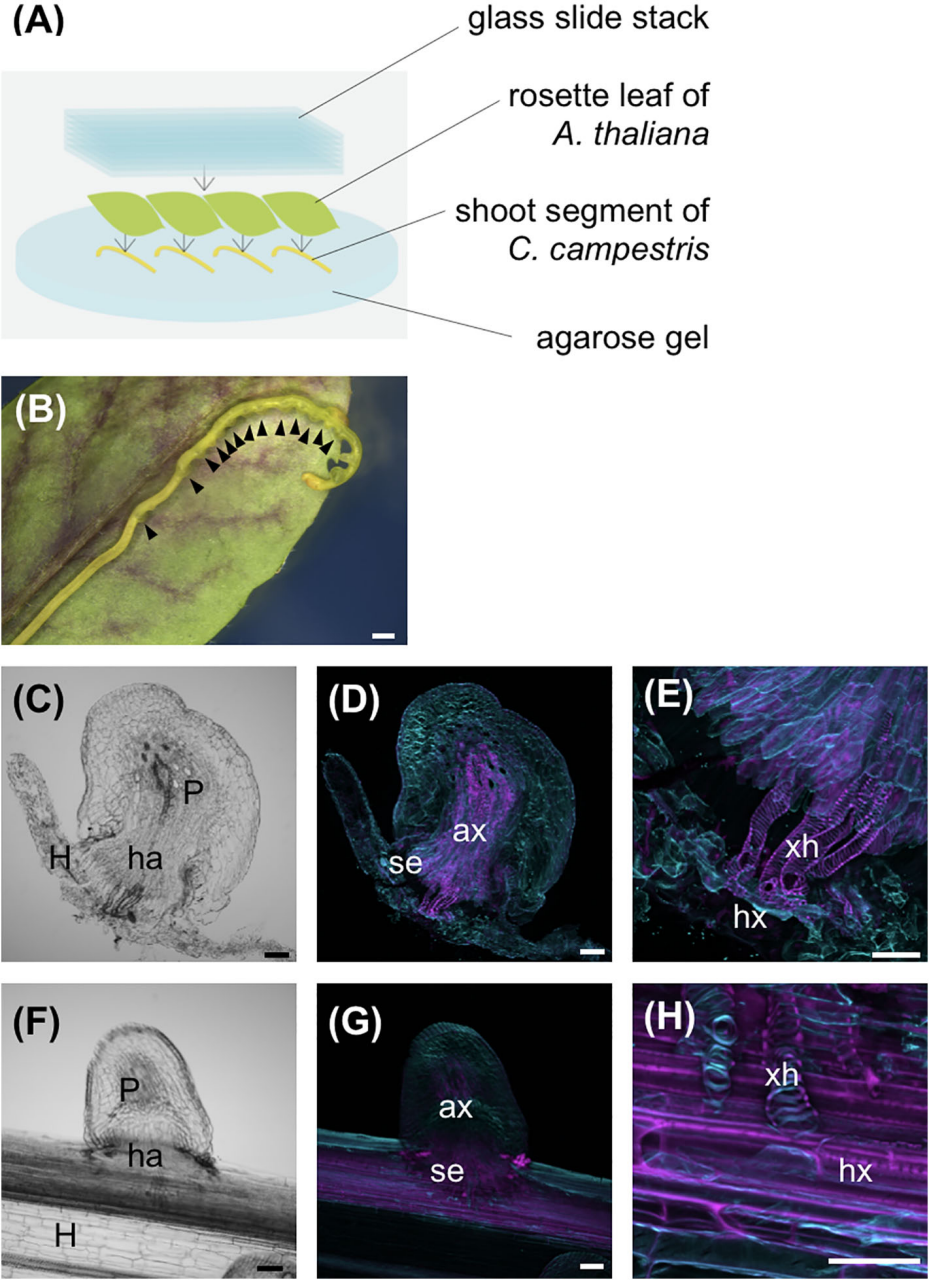
Host xylem is necessary for search hyphae to differentiate into xylem vessel cells. **(A)** Schematic of the *in vitro* haustorium induction procedure with *A. thaliana* rosette leaves used as the host. Excised *C. campestris* lateral shoots were covered with *A. thaliana* rosette leaves and pressure applied with a stack of glass slides. **(B)** Formation of haustoria in an excised *C. campestris* lateral shoot under an *A. thaliana* rosette leaf and a stack of glass slides. Black arrowheads indicate haustoria. **(C–E)** Images of a haustorium produced by the *in vitro* induction system with host rosette leaf at the stage of 96 hours after induction. **(C)** A bright-field image of a longitudinal section of the haustorium. **(D, E)** Digital accumulation fluorescence images of Z-serial optical sections of the same longitudinal section as for **(D)**, which was stained with Fluostain I and propidium iodide. **(E)** A high-magnification image of **(D)**. **(F–H)** Images of a haustorium from *C. campestris* parasitizing an intact inflorescence stem of *A. thaliana* at the stage of 66 hours after coiling. **(F)** A bright-field image of a longitudinal section. **(G, H)** Digital accumulation fluorescence images of Z-serial optical sections of the same longitudinal section as for (F), which was double stained with Fluostain I and propidium iodide. **(H)** A high-magnification image of **(G)**. Scale bars: **(B)** 1 mm; **(D–H)** 100 μm. P, parasitic plant; ha, haustorium; ax, axial cell; se, search hypha; xh, xylem hypha; hx, host xylem; H, host plant.

Lateral shoots of *C. campestris* stem were cut 3 cm below the apex. Shoot segments were placed on 3% agarose gel containing 0.1% Plant Preservation Mixture™ (Plant Cell Technology, Inc.), weighted with a stack of glass slides (S1225, Matsunami Glass Ind., Ltd.), and incubated under blue light irradiation at 25°C (**Figure 1A**). To induce differentiation of search hyphae into xylem hyphae, a 3-cm-long lateral shoot segment of *C. campestris* was overlaid with a fresh rosette leaf of 4–5-week-old *A. thaliana*, and was weighted with a stack of glass slides (**Figure 2A**). Haustoria were classified into two types: haustoria protruding search hyphae were designated as true haustoria, while conical-shaped ones were designated as pseudo haustoria according to Hong et al. (2011). The numbers of true and pseudo haustoria were counted under a stereomicroscope (M205 FA, Leica).

### Phytohormone Treatment of Search Hyphae

*C. campestris* lateral shoot sections that had been pressed for 54 hours to induce haustoria were placed on 3% agarose media containing different phytohormone compositions. Sections were placed so that search hyphae were in contact with the medium and were incubated in a growth chamber for 48 hours. Shoot segments were incubated under the same conditions as for the *in vitro* haustorium induction system. Phytohormone compositions in the media were as followed: (1) 1 μM brassinolide (BL) and 10 mM H_3_BO_3_; (2) 0.1 mg/L naphthaleneacetic acid (NAA) and 0.2 mg/L benzyladenine (BA); (3) 1.25 mg/L 2,4-dichlorophenoxyacetic acid (2,4-D), 0.25 mg/L kinetin and 10 μM Bikinin; and (4) 50 ng/ml kinetin, 500 ng/ml 2,4-D and 1 mM BL.

### Histological Staining and Microscopy

Lateral shoots with induced haustoria were embedded in 5% agarose gel and sectioned transversely or longitudinally at a thickness of 60 μm using a vibratome (VT1200S, Leica). Sections were fixed with FAA solution (4% paraformaldehyde, 20 mM sodium cacodylate buffer) and stored at 4°C. Sections were cleared with an ethanol series (50%, 60%, 70%, 80%, 90%, and 95%) and washed three times with phosphate buffered saline (PBS). The cleared sections were stained with a solution containing 0.002% Fluostain I (Sigma-Aldrich) and 0.2% propidium iodide (Fujifilm Wako Pure Chemical) for 1 hour followed by washing three times with PBS. The stained sections were immersed in 50% 1 × PBS/glycerol solution, and Z-serial optical sections were obtained under a laser scanning confocal microscope (FV1000-D, Olympus). Digital accumulation of Z-serial optical sections was performed using ImageJ (ver. 2.0.0).

Images of *C. campestris* shoots with induced haustoria were obtained using a stereomicroscope (for **Figures 1B** and **2B**). Tissues from which RNA samples for RNA-sequencing (RNA-seq) analysis were prepared were visualized using a stereomicroscope (for **Figures 4A–C**), or a light microscope (DM RXP, Leica) after transverse (for **Figure 4D**, control) or longitudinal (**Figure 4D**, 57 and 87 hours after induction) sectioning.

### Transcriptome analysis of *In Vitro* Haustorium Development

Tissues for RNA extraction were manually excised from control and *in vitro* induced-haustorium shoots under a stereomicroscope. Control samples were excised from epidermal and cortical tissues obtained at 0 hours after induction (hai) from 3-cm shoot sections that had not been pressed by glass slides or placed in contact with host tissue [0 hai (−/−)]. For induced samples, haustorium development was induced in 3-cm lateral shoot sections of *C. campestris* as described above. Sections were pressed under a stack of glass slides with or without contact with host leaf tissue for 57 or 87 hours after induction (**Figure 4**). Pressed samples with host contact were designated 57 hai (+/+) and 87 hai (+/+), and pressed samples without host contact were designated 57 hai (+/−) and 87 hai (+/−). Haustoria for RNA extraction were manually excised from shoot segments, with minimal host tissue included for the 57 hai (+/+) and 87 hai (+/+) samples.

Tissue samples were immediately frozen in liquid nitrogen, and total RNAs were isolated using an RNeasy Plant Mini Kit (Qiagen Inc.) with RNase-Free DNase (Qiagen Inc.) according to the manufacturer's protocol. Extracted RNA was quantified with a NanoDrop spectrophotometer (ND-1000, Thermo Fisher Scientific). RNA quality was assessed with an Agilent 2100 Bioanalyzer using an RNA 6000 Nano Kit (Agilent Technolozies, Inc.).

RNA-seq was performed using the BGISEQ-500 platform (BGI), and 100 bp pair-end reads for each library were mapped independently to the references described below using the HISAT2 (ver. 2.1.0) alignment program (Kim et al., 2015). Annotated reference genome sequences for *C. campestris* were downloaded from plaBiPD (https://www.plabipd.de) (Vogel et al., 2018). Three biological repeats were used for reference genome mapping. One of the three 87 hai (+/+) treatment libraries was an outlier according to hierarchical clustering and was therefore excluded from differential expression analysis. Transcript expression levels and differentially expressed genes (DEGs) were determined using the StringTie (ver. 1.3.6; Pertea et al., 2015) and TCC (Sun et al., 2013) packages, respectively. Transcript expression levels were normalized to transcripts per million (TPM), and genes with q-value < 0.01 were regarded as DEGs.

### Transcriptome Analysis of Haustorium Development in *C. campestris* Parasitizing Intact *A. thaliana*

Coiling regions of *C. campestris* lateral shoots parasitizing an intact *A. thaliana* inflorescence stem were harvested 0, 12, 42, and 54 hac. The harvested tissues obtained at 0 hac consisted of epidermis and cortex from the concave region of the parasite stem. Harvested tissue at 12 hac contained prehaustoria and those obtained at 42 hac and 54 hac contained haustoria. Tissue samples were transverse sectioned (100 μm) to the host stem axis using a vibratome. Haustorial regions were excised from transverse sections by laser microdissection using a PALM MicroBeam (Carl Zeiss Microscopy GmbH) (**Supplementary Figure 3**). Control samples were derived from *C. campestris* lateral shoots that were irradiated with blue light for 24 hours, but which did not coil around the host stem. Control samples consisted of the epidermis and cortex and were harvested, sectioned and subjected to the laser micro-dissection as coiled samples.

Excised tissue samples were immersed in RNAlater Solution (Thermo Fisher Scientific) and stored at 4°C. Total RNAs were isolated using an RNeasy Plant Mini Kit with RNase-Free DNase according to the manufacturer's protocol. Extracted RNA was quantified with and a NanoDrop spectrophotometer. RNA quality was assessed with an Agilent 2100 Bioanalyzer (Agilent Technolozies, Inc.) using an RNA 6000 Pico Kit (Agilent Technolozies, Inc.). For screening, cDNA libraries were constructed using an NEBNext RNA Library Prep Kit for Illumina (NEW ENGLAND BioLabs), according to the manufacturer's protocol. After ligation of indexed adaptors (**Supplementary Table 1**), products were purified using Agenocourt AMPure XP Beads (Beckman Coulter) and amplified by PCR with KAPA Hifi HotStart ReadyMix (KAPA Biosynthesis). The cDNA libraries were separated by 2% agarose gel electrophoresis, extracted using a QIAquick Gel Extraction Kit (QIAGEN), and finally quantified using a Library Quantification Kit (Takara, Japan). In total, 15 cDNA libraries consisting of three biological replicates of five experimental conditions (0 hac, 12 hac, 42 hac, 54 hac, and control) were pooled in equal amounts (18 pM and 20 pM) for multiplexing. Libraries were sequenced using a Genome Analyzer IIx instrument (Illumina), and the 33 nt single-end reads from each library were mapped independently to the references described above using the HISAT2 (ver. 2.1.0) alignment program (Kim et al., 2015). Three biological replicates were used for reference genome mapping. Transcript expression levels and DEGs were determined using the StringTie (ver. 1.3.6; Pertea et al., 2015) and TCC (Sun et al., 2013) package, respectively. Transcript expression levels were normalized to TPM, and genes with q-value < 0.01 were regarded as DEGs.

### Phylogenetic Analysis

Similarity searches were performed against The Arabidopsis Information Resource 10 database (TAIR10; ftp://ftp.arabidopsis.org/home/tair/Genes/TAIR10_genome_release/TAIR10_gff3/TAIR10_GFF3_genes.gff; Lamesch et al., 2012) using BLASTP. Collected protein sequences were aligned using MAFFT (ver. 7.427) (Kato and Standley, 2013) then visually inspected and manual refined. Gaps and ambiguous sites were removed from the alignment. Phylogenetic trees were constructed with a maximum likelihood method using MEGA7 (Kumar et al., 2016) with bootstrap replication of 1,000.

### Clustering Analysis

Soft clustering was performed on gene sets that were defined as DEGs using Mfuzz (Futschik and Carlisle, 2005) based on TPM. Functional annotations of DEGs and clustered gene sets were produced from the reference annotation information.

### Enrichment Analysis

Enrichment was determined using the hypergeometric distribution (Johnson et al., 1992) and Benjamini-Hochberg procedure (Benjamini and Hochberg, 1995).

## Results

### Establishment of an *In Vitro* System for Induction of Haustorium Development in *C. campestris*

An *in vitro* system for inducing haustorium development outside an intact host was developed and used to examine the host-dependent formation of haustoria in *C. campestris*. Previous studies reported that tactile stimuli induced the formation of haustoria under far-red light irradiation (Tada et al., 1996; Olsen et al., 2016), and that blue light irradiation promoted parasitism in *Cuscuta* seedlings (Lane and Kasperbauer, 1965). Accordingly, in this study, the relationship between haustorium formation and a mechanical stimulus was investigated by pressing lateral shoot segments of *C. campestris* with a stack of glass slides under blue light irradiation for 72 hours (**Figure 1A**). Two types of haustoria were induced using this experimental system. One is those protruding search hyphae, which we termed true haustoria, and the other is conical-shaped one, which we termed pseudo haustoria (Hong et al., 2011) (**Figure 1B**). True haustoria accounted for approximately 30% of observed haustoria when one glass slide was used to apply pressure to the lateral shoot sections (equivalent to approximately 20.74 kPa), whereas about 75% of haustoria protruded search hyphae when seven glass slides were used to apply pressure of ~145.20 kPa (**Figure 1C**). However, the number of slides used to apply pressure had no significant effect on the overall number of haustorium produced by the lateral shoots (**Figure 1C**). Under these conditions, when *C. campestris* shoot segment did not attach to the host, elongation of axial cells and search hyphae was observed in the true haustoria, search hyphae did not differentiate into xylem hyphae (**Figures 1D–F**). These true haustoria were similar to those observed just after penetration into host inflorescence stems (**Figures 1G–I**).

Next, to investigate the involvement of host-derived phytohormones in haustorium development, excised shoots with induced true haustoria were placed on solid agarose media containing four different phytohormone or chemical mixtures, that reportedly induced xylem vessel differentiation in other angiosperms (Demura et al., 2002; Kubo et al., 2005; Kondo et al., 2016; Tan et al., 2018). Shoots were placed to ensure that search hyphae were in contact with the agarose medium. No visible alterations in haustorium development were observed after exposure to the phytohormone mixtures under the conditions we examined (**Supplementary Figure 1**).

As discussed above, although haustorium development in *C. campestris* proceeded to the host-penetration stage upon application of pressure and exposure to blue light irradiation, search hyphae did not differentiate into xylem hyphae. Three typical phytohormones that were previously shown to play essential roles in xylem vessel formation in other angiosperms were not effective in inducing differentiation of search hyphae into xylem hyphae. These results suggest that the inability to induce differentiation in the absence of host tissue is not due to a lack of phytohormones derived from the host plants, implying that host-derived factors other than auxins, cytokinins, or brassinosteroids are needed for xylem vessel differentiation of *Cuscuta* haustorial cells.

### Differentiation of Search Hyphae Into Xylem Hyphae is Induced Upon Contact With Host Xylem

To determine whether search hyphae produced by the *in vitro* haustorium induction system had the potential to differentiate into xylem hyphae upon contact with host xylem, lateral shoot segments of *C. campestris* were overlaid with fresh *A. thaliana* rosette leaves, and then pressed with a stack of glass slides (**Figure 2A**). Haustorium invaded the host tissue in the presence of host leaves. Helical-patterned SCW differentiation was observed where the search hyphae came into contact with the host xylem (**Figures 2B–E**). The SCW deposition pattern of xylem hyphae observed with the *in vitro* induction system was comparable to the that observed during *C. campestris* parasitization of intact host plant stems (Dawson et al., 1994) (**Figures 2F–H**). These results indicate that the search hyphae produced by the *in vitro* induction system have the same potential for differentiation as the cells that penetrate intact host stems and eventually differentiate into xylem vessel cells during parasite-host interactions.

**Figure 3:**
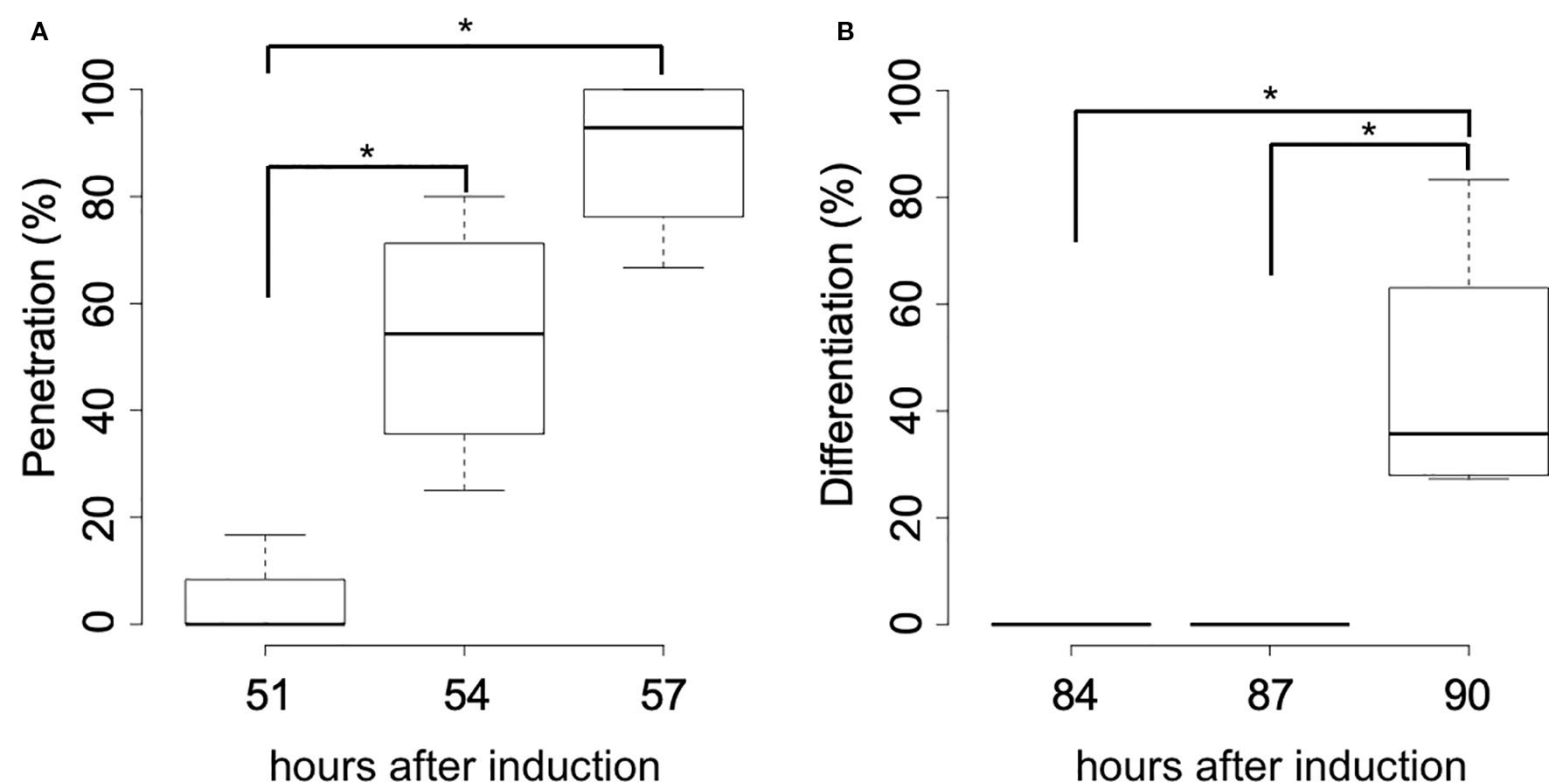
Time-course of the haustorial penetration and differentiation of search hyphae. **(A)** Percentage of haustoria that penetrated *A. thaliana* rosette leaves at the stage of 51, 54, and 57 hours after induction. **(B)** Percentage of haustoria whose search hyphae differentiated into xylem hyphae at the stage of 84, 87, and 90 hours after induction. Significance was determined by the Wilcoxon rank-sum test (n = 4). Asterisks indicate significant differences in pairwise comparisons (p < 0.05).

Subsequent time-course observation of the *in vitro* induction process revealed that most haustoria had penetrated the host rosette leaf by 57 hai (**Figure 3A**), and that differentiation of search hyphae into xylem hyphae had occurred by 90 hai (**Figure 3B**). Thirty hours after penetration, xylem vessel cells had formed in the induced haustoria.

### Transcriptional Regulation During Haustorium Penetration of Host Tissue

**Figure 4:**
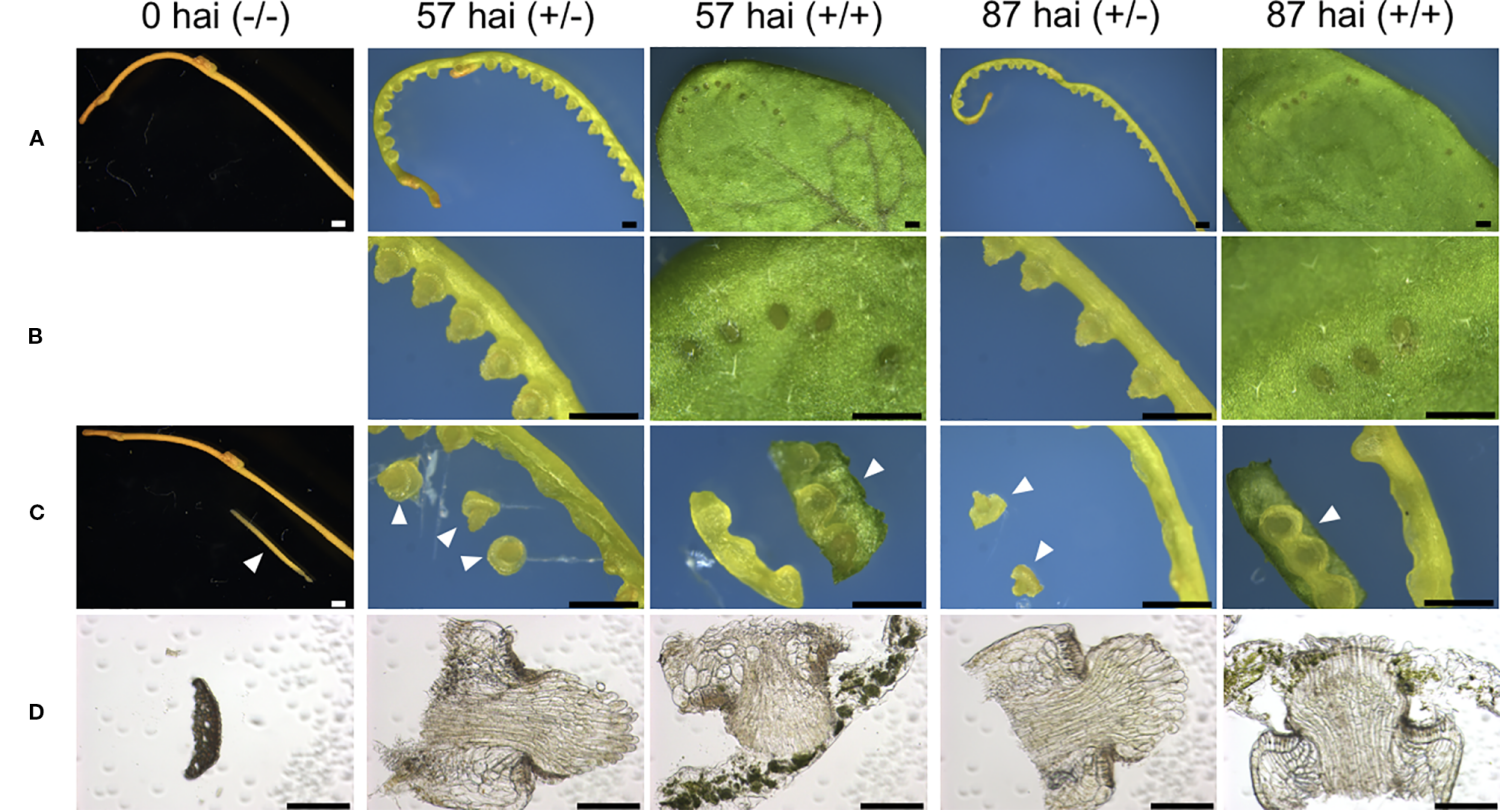
Tissue sampling at different stages of *in vitro* haustorium development for RNA-seq libraries. **(A)** Control sample of intact lateral shoot of *C. campestris* at the onset of the induction is designated here as 0 hai (−/−). Three-centimeter long segments prepared from the lateral shoots were subjected to *in vitro* haustorium induction under pressure from a stack of glass slides under blue light (440 nm) irradiation at the dosage of 7 μmol m^−2^ s^−1^, in the absence (+/−) or presence (+/+) of an *A. thaliana* rosette leaf for 57 or 87 hours after induction (hai). These samples are designated here as 57 hai (+/−), 57 hai (+/+), 87 hai (+/−), and 87 hai (+/+), respectively. **(B)** Magnified images of **(A)**. **(C)** Control sample, 0 hai (−/−), consisted of epidermal and cortical cells isolated from lateral shoot segments. Samples of 57 hai (+/−) and 87 hai (+/−) consisted of haustoria excised from shoot segments, while samples of 57 hai (+/+) and 87 hai (+/+) consisted of shoot segments containing haustoria with minimal host leaf included. Arrowheads show samples used for individual RNA-seq libraries. **(D)** Bright-field images of a transverse section for 0 hai (−/−), and longitudinal sections for the other four samples used for RNA-seq. Scale bars: **(A–C)** 1 mm; **(D)** 200 μm. hai, hours after induction.

**Figure 5:**
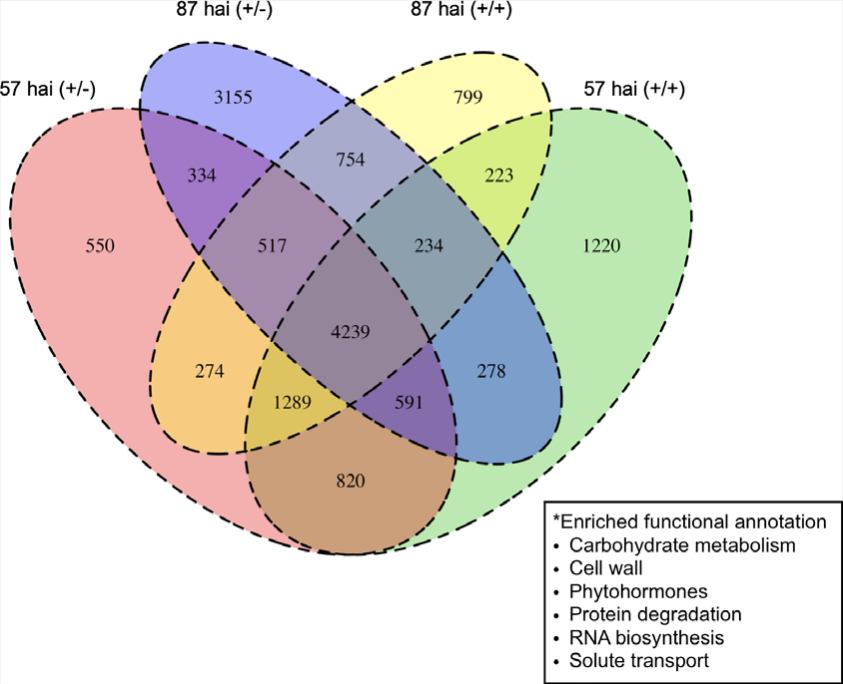
Numbers of differentially expressed genes up-regulated in the haustorium at 57 or 87 hours after induction (hai) when subjected to pressure in the absence (+/−) or presence (+/+) of host tissue compared with control tissue containing epidermal and cortical cells at 0 hai (−/−). Enriched functional annotations for all four haustorial conditions are shown in the box. hai, hours after induction.

**Figure 6:**
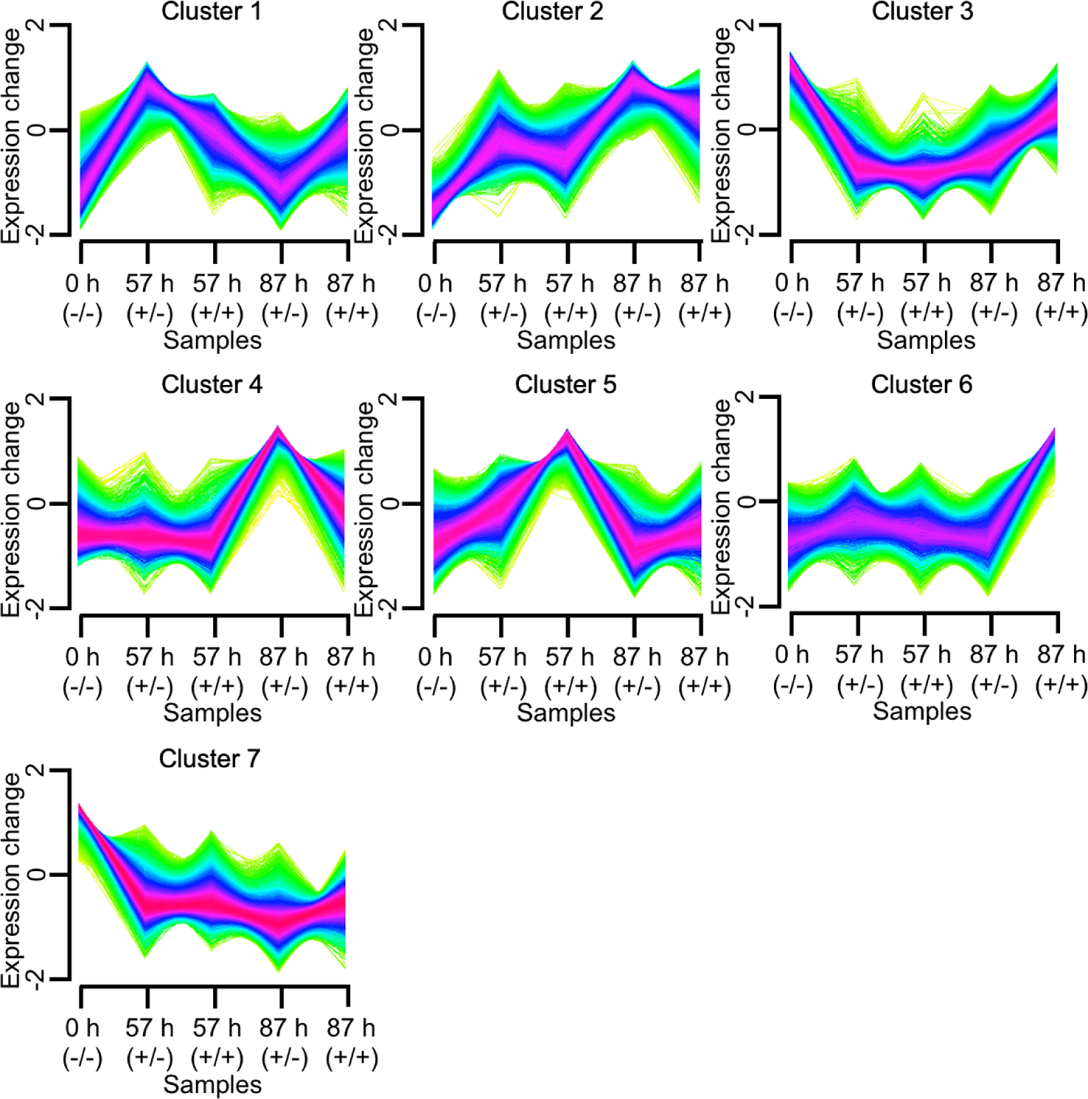
Clustering analysis of 28,958 differentially expressed genes (false discovery rate < 0.01) using Mfuzz. Tissue samples were taken 57 or 87 hours after induction with pressure in the absence (+/−) or presence (+/+) of an *A. thaliana* rosette leaf, and designated here as 57 h (+/−), 57 h (+/+), 87 h (+/−), and 87 h (+/+), respectively. For control, samples containing epidermal and cortical cells of *C. campestris* stem were taken at the onset of the induction and designated here as 0 h (−/−). h, hours after induction.

RNA-seq was used to examine the transcriptional regulation of haustorium development during penetration into host tissue, RNA-seq libraries were prepared from tissue samples taken at specific time points determined through time-course observation of haustorium development (**Figure 3**). Tissue samples were derived from haustoria that penetrated into the host tissue at 57 hai (+/+) and at 87 hai (+/+), haustroria that did not contact host tissue at 57 hai (+/−) and at 87 hai (+/−), and epidermal and cortical cells of *C. campestris* at 0 hai (−/−) as a no-haustorium control (**Figure 4**). Sequenced read pairs were mapped against the *C. campestris* genome. The mapping rate of the 57 hai (+/+) and 87 hai (+/+) sequence reads was 16.6% lower than reads from the other libraries (**Supplementary Figure 2**). The 57 hai (+/+) and 87 hai (+/+) libraries contained reads derived from host tissues, suggesting that the lower mapping rate was due to the proportion of reads that did not map to the *C. campestris* reference genome. Differential expression analysis using a false discovery rate (FDR) < 0.01 produced 15,277 DEGs in the haustorium compared with the epidermal and cortical cells (**Supplementary Data 1****–****4**). Of these 4,239 DEGs, 1,721 of which were functionally annotated, were shared among the four haustorium conditions (**Figure 5**). Consitent with previous gene expression studies of the genus *Cuscuta* (Ranjan et al., 2014; Olsen et al., 2016), genes encoding functionally annotated proteins for carbohydrate metabolism, cell wall, and solute transport, as well as phytohormones, protein degradation, and RNA biosynthesis (*q*-value < 0.01, **Figure 5**) were up-regulated in the haustorium. DEGs were also compared among all five conditions (including the no-haustorium control), using an FDR < 0.01, and 28,958 DEGs were identified. After normalizing the count data to TPM, DEGs were soft-clustered, and clusters were analyzed for enriched functional annotations (**Figure 6**, **Table 1**, and **Supplementary Data 5**). Of the 28,958 DEGs, 11,802 genes were functionally annotated in reference annotation data of *C. campestris*. At 57 hai (+/+), genes encoding proteins for cell wall, phytohormones, protein modification, and secondary metabolism were significantly enriched (*q*-value < 0.01, **Figure 6**, **Table 1**, and **Supplementary Data 5**). At 87 hai (+/+), phytohormones, polyamine metabolism, and RNA biosynthesis were up-regulated (*q*-value < 0.01, **Figure 6** and **Table 1**, **Supplementary Data 5**). These results indicate that gene expression is dynamically regulated by penetration into host tissue as well as during haustorium formation.

**Table 1:** Enriched functional annotation in each of the seven clusters of DEGs.

Cluster No.	Functional annotation	q-value
1	Cell wall	8.99E-03
1	Lipid metabolism	2.32E-03
1	Nutrient uptake	4.32E-03
1	Protein modification	7.84E-08
1	Solute transport	1.78E-04
2	Protein degradation	1.37E-05
2	RNA biosynthesis	3.25E-08
2	Solute transport	7.86E-03
3	Amino acid metabolism	1.53E-05
3	Cell cycle	1.09E-27
3	DNA damage response	2.46E-04
3	Environmental stimuli response	1.11E-03
3	Nucleotide metabolism	1.57E-04
3	Photosynthesis	4.17E-10
3	Protein biosynthesis	8.41E-73
3	Protein translocation	8.24E-21
3	RNA processing	6.78E-36
4	Carbohydrate metabolism	3.86E-03
4	Coenzyme metabolism	8.47E-05
4	Photosynthesis	1.18E-32
4	Secondary metabolism	3.85E-03
5	Cell wall	4.56E-12
5	Phytohormones	3.73E-03
5	Protein modification	3.48E-06
5	Secondary metabolism	5.05E-04
6	Phytohormones	3.22E-04
6	Polyamine metabolism	3.22E-04
6	RNA biosynthesis	1.82E-09
7	Cell wall	4.45E-06
7	Cellular respiration	3.53E-25
7	Cytoskeleton	1.76E-04
7	Lipid metabolism	2.21E-09
7	Membrane vesicle trafficking	2.18E-07
7	Protein biosynthesis	1.48E-18

Significance was determined by the hypergeometric distribution (p-value < 0.01) and Benjamini-Hochberg procedure (q-value < 0.01).

### Transcriptional Regulation of Xylem Cell Differentiation in Haustoria

**Figure 7:**
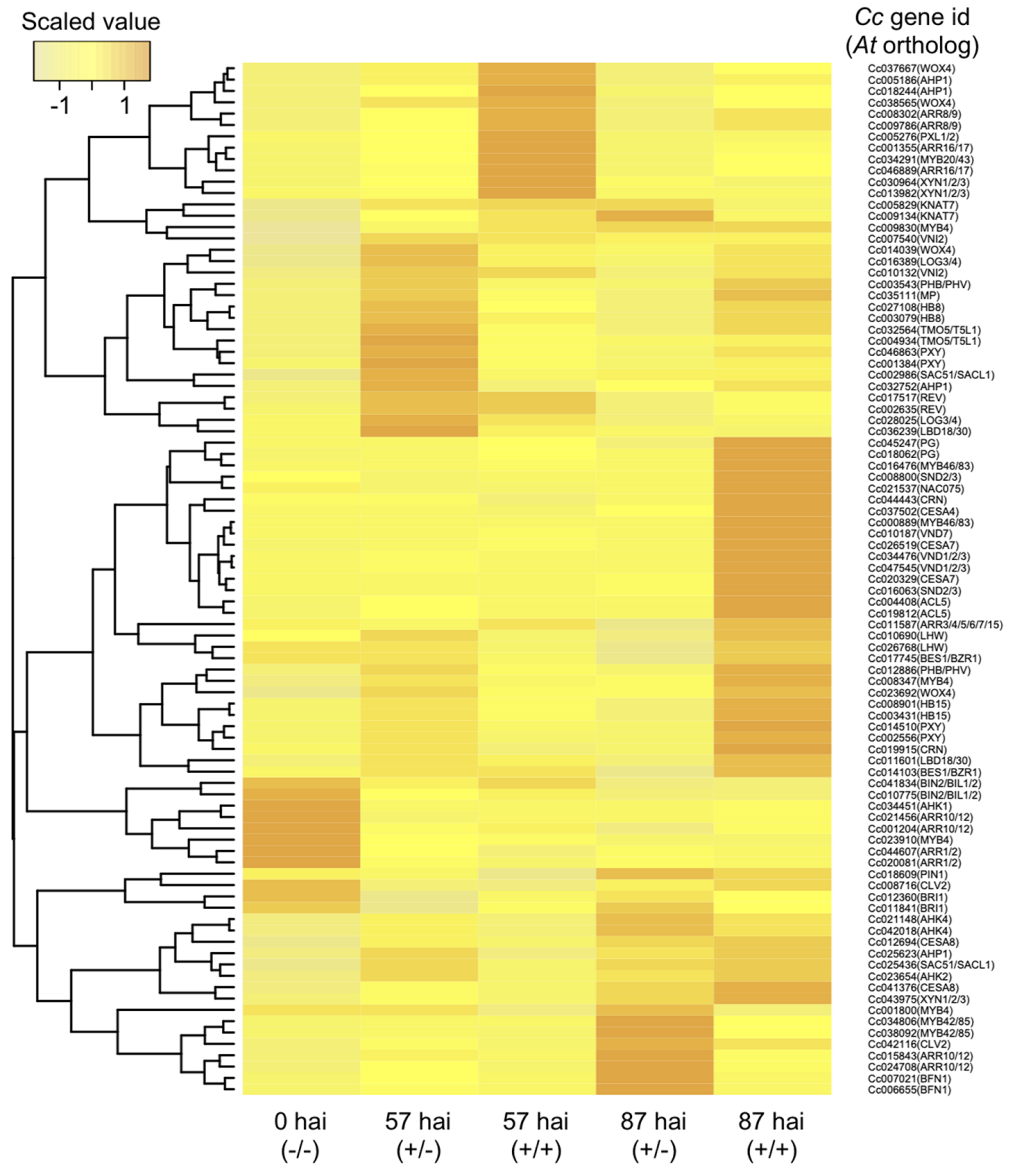
Hierarchical clustering of *C. campestris* orthologous genes with biological functions putatively related to vascular development in *A. thaliana*. Transcript per million data from RNA-seq results of the *in vitro* induction system were normalized, and relative gene expression levels among the five samples are visualized in a heat map image according to the color scale shown in the left-hand panel. Samples were taken 57 or 87 hours after induction (hai) with pressure in the absence (+/−) or presence (+/+) of an *A. thaliana* rosette leaf and designated here as 57 hai (+/−), 57 hai (+/+), 87 hai (+/−), and 87 hai (+/+), respectively. For control, samples containing epidermal and cortical cells of *C. campestris* stem were taken at the onset of the induction and designated as 0 hai (−/−). hai, hours after induction.

Contact with host xylems was necessary for differentiation of search hyphae into xylem hyphae, and we therefore focused on relevant genes whose expression profiles correlated to vascular development during the penetration of host tissue. RNA-seq data for orthologous genes reported to be involved in the development and proliferation of vascular stem cells were examined. At 57 hai (+/−), Cc*MP* (Cc035111), *Cc*TMO5 and *CcT5L1* (Cc004934 and Cc032564)*, CcLHW* (Cc010690 and Cc026768)*, CcLOG3* and *CcLOG4* (Cc028025 and Cc016389), and *CcHB8* (Cc027108 and Cc003079) were all up-regulated (**Figure 7**), indicating that haustoria acquired the potential for differentiation into xylem cells in the absence of penetration. The results indicate that vascular stem cells and xylem precursor cells can differentiate within the *Cuscuta* haustorial tissue without penetration into the host tissue.

It should also be noted that some members of the type-A *ARABIDOPSIS RESPONSE REGULATORs* (Cc008302, Cc009786, Cc001355, and Cc046889), which might negatively regulate cytokinin signaling, are up-regulated at 57 hai (+/+). This might suggest that the vascular stem cell proliferation is repressed *via* cytokinin signaling in the haustorium after the penetration into the host tissue (**Figure 7**).

*CcVND7* (Cc010187), the orthologous gene to *VND7*, was up-regulated at 87 hai (+/+), but no ortholog of *VND6* was identified (**Figure 7**). Genes active downstream of *VND7*, namely, *MYB46*, *MYB83* (Cc016476 and Cc000889), *CELLULOSE SYNTHASE A4/IRREGULAR XYLEM 5* (*CESA4/IRX5)* (Cc037502), and *CESA7/IRX3* (Cc020329 and Cc026519), exhibited the same expression pattern as *CcVND7* (Kubo et al., 2005) (**Figure 7**). In *A. thaliana*, CESA4 and CESA7 are involved in synthesis of SCWs (Taylor et al., 2000; Taylor et al., 2003), with two functionally redundant MYB transcription factors, MYB46 and MYB83, acting as master regulators of SCW biosynthesis (Zhong et al., 2007; McCarthy et al., 2009). Here, two lignin biosynthesis-related genes, four genes encoding cysteine peptides, and eleven genes encoding serine peptidase were identified in Cluster 6 (**Figure 6**).

On the other hand, at 87 hai (+/−), the expressions of *BIFUNCTIONAL NUCLEASE 1* (Cc007021 and Cc006655), *CcARR10* and *CcARR12* (Cc015843 and Cc24708) were up-regulated, but activation of genes encoding proteins involved in promotion of xylem vessel cell formation was not found (**Figure 7**).

**Figure 8:**
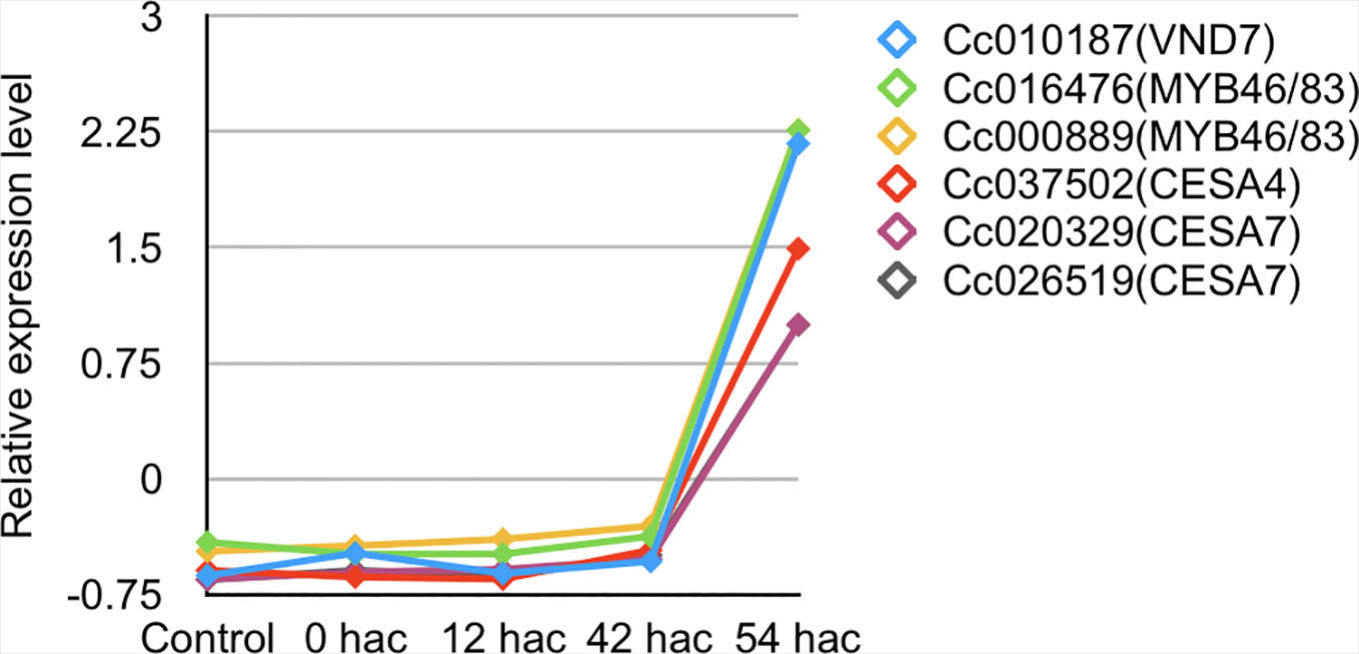
Expression patterns of *C. campestris* orthologous genes related to xylem vessel cell differentiation in *A. thaliana*. Transcripts per million data from RNA-seq analysis of haustorium development in *C. campestris* parasitizing an intact host were normalized. Samples for RNA-seq analysis were collected from transverse sections of haustoria at a thickness of 100 μm using laser microdissection. Control, tissue region containing epidermal and cortical cells of *C. campestris* after irradiation with blue light for 24 hours without contact with the host; 0 hac, tissue region consisting of epidermal and cortical cells of the contact site with the host inflorescence stem just after coiling; 12 hac, prehaustorium at the stage of 12 hours after coiling; 42 hac and 54 hac, haustoria penetrating into the host plant at the stage of 42 and 54 hours after coiling. hac, hours after coiling.

Importantly, a set of genes for xylem vessel cell formation whose expression were up-regulated at the stage of 87 hai (+/+) in the *in vitro* system were also up-regulated in the haustorium produced in *C. campestris* shoot 54 hours after coiling around an intact inflorescence stem of *A. thaliana*, when search hyphae contacted the host xylem (**Figure 8** and **Supplementary Figure 3**). Thus, the expression patterns of these genes in *in vitro* system were consistent with those in the haustorium development in *C. campestris* shoot parasitizing an intact host. These findings indicate that contact of search hypha with the host xylem triggers the up-regulation of a *VND7* ortholog in *C. campestris* and induces the formation of xylem vessel cells in the haustorium.

## Discussion

In this study, an *in vitro* system for inducing *C. campestris* haustorium formation through application of pressure in the presence or absence of host tissue was developed used to analyze host-dependent transcriptional regulation during haustorium development. Two types of haustoria, true haustorium and pseudo haustorium, were induced in *C. campestris* lateral shoots in the absence of host plants. The ratio of true haustorium to pseudo haustorium was dependent on the pressure applied to the *C. campestris* shoots. Application of a force of 145.20 kPa to a single 3-cm segment of *C. campestris* lateral shoot was optimum for the effective formation of true haustoria.

Our findings are consistent with previous research showing that *Cuscuta* plants promote haustorium development by sensing the pressure generated by coiling around the stem of a host plant (Lee, 2009). Our results suggest that the coiling of *C. campestris* around the host plant might exert pressure at a load of more than 100 kPa.

The *in vitro* pressure-based system was sufficient to induce elongation of search hyphae and axial cells, but was not sufficient to promote differentiation into xylem vessel cells. These results suggest that additional signaling derived from the host plant is necessary for xylem differentiation in *C. campestris* haustoria.

Next, *in vitro*-induced haustoria were cultivated on solid agarose media containing phytohormones that were previously shown to induce differentiation into xylem vessel cells (Demura et al., 2002; Kubo et al., 2005; Kondo et al., 2016; Tan et al., 2018); however, this exposure did not stimulate the haustorial cell differentiation into xylem vessel cells in *C. campestris*. These results suggest that the host-derived signaling factors that trigger differentiation of search hyphae into the xylem cells are not phytohormones.

Auxin activates MP transcription factor during vascular development in angiosperms. MP enhances ATHB8 expression and cytokinin biosynthesis, which promote vascular stem cell development and proliferation (Scarpella et al., 2006; Ohashi-Ito et al., 2014). In addition, brassinosteroids promote the transcription of HD-ZIP III transcription factor family genes, which play key roles in the establishment of vascular patterning in *Zinnia elegans* (Ohashi-Ito et al., 2002; Carlsbecker and Helariutta, 2005). This study examined transcriptional regulation of haustorium development in *C. campestris* and found that *MP* and downstream genes related to vascular stem cell specification and proliferation were up-regulated in the haustorium, even in the absence of host plant tissue. It is therefore likely that the host-derived signaling factor(s) capable of triggering differentiation from search hyphae into xylem hyphae is those that activate the process of xylem vessel cell differentiation after vascular stem cell fate determination.

Previous morphological analyses showed that search hyphae penetrating into the host xylem differentiated into xylem hyphae (Hong et al., 2011). This suggests that contact between search hypha and the host xylem is required for the differentiation of search hyphae into xylem hyphae and also suggests that search hyphae might receive signals as a result of contact with the host xylem. The *C. campestris* orthologs of *VND7*, *MYB46*, and *MYB83* were expressed after search hyphae contacted the host xylem. These transcription factors are master regulators of xylem vessel cell differentiation and SCW biosynthesis (Kubo et al., 2005; Zhong et al., 2007; McCarthy et al., 2009). These data suggest that search hyphae receive host-derived signals that activates transcription of *CcVND7* and stimulate differentiation into xylem hyphae in a non-cell-autonomous manner. Furthermore, the transcriptome data showed that the *NAC DOMAIN CONTAINING PROTEIN 75* (*NAC075*) ortholog in *C. campestris* was also up-regulated. NAC075 is thought to regulate the expression of *VND7* in *A. thaliana* (Endo et al., 2015), and the parasitic plant might therefore recognize signals from the host plant that promote *CcNAC075* expression.

This study characterized the dynamics of transcriptional regulation during the differentiation of xylem vessel cells in haustorium development in *C. campestris*. The results suggest that haustoria have acquired the potential for differentiation into xylem vessel cells without penetration into host tissue, probably through activation of genes involved in vascular stem development and proliferation. However, the expression of genes needed for xylem vessel cell differentiation appears to require contact between search hypha and the host xylem. This contact might be critical for efficient establishment of the xylem connection between the host plant and the parasitic plant. Signals derived from the host xylem appear to trigger the differentiation into xylem hyphae, possibly through the expression of *CcVND7*, regulated by CcNAC075, in the search hyphae. Further research is needed to identify host-derived signaling factors and signal transduction pathways that regulate expression of *CcNAC075* and *CcVND7* during parasitic invasion.

## Data Availability Statement

The RNA-seq data presented in this study have been deposited with links to accession number DRA009453 in the DDBJ database (Sequence Read Archive: https://trace.ddbj.nig.ac.jp/dra/index_e.html).

## Author Contributions

YK performed the experiments and data acquisition with occasional discussions with RY, RS, MO, TD, TK, and NS under supervision and organization of KN. The manuscript was written by YK under supervision of KN with discussion with YR, RS, TD, MO, NS, and TK. Deposition of sequence data was supported by RS.

## Funding

This work was supported in part by Grants-in-Aid for the Japan Society for the Promotion of Science (JSPS) fellows (KAKENHI Grant Number 17J03964 to YK), for Challenging Research (17K19374 to KN) and for Scientific Research on Innovative Areas (24114005 to KN and JP18H05484 and 18H05489 to TD) from JSPS.

## Conflict of Interest

The authors declare that the research was conducted in the absence of any commercial or financial relationships that could be construed as a potential conflict of interest.

## Supplementary Material

Supplementary Table 1 Sequences of indexing primers.

Supplementary Figure 1 Haustoria produced by the *in vitro* induction system were further incubated on a solid agarose medium containing phytohormones/chemicals mixtures for 48 hours. Digital accumulation images of Z-serial optical sections of the haustorium. Scale bars; 100 μm. BL, brassinolide; NAA, naphthaleneacetic acid; BA, benzyladenine; 2,4D, 2,4-dichlorophenoxyacetic acid.

Supplementary Figure 2 Mapping counts of reads for each library against the *C. campestris* genome.

Supplementary Figure 3 Tissue samples from haustorium development in an intact host for RNA-sequencing libraries. Samples for each stage of the haustorium development were collected from transverse sections of 100 μm thickness using laser microdissection. Outside of the green circle showed the collected region for the control sample. Inside of the green circles showed the collected regions at 0, 12, 42, and 54 hours after coiling (hac). control, epidermal and cortical cells of *C. campestris* shoot irradiated with blue light for 24 hours; 0 hac, epidermal and cortical cells of the contact site with the host inflorescence stem just after the coiling; 12 hac, prehaustorium; 42 hac, haustorium just after penetration into the host; 54 hac, haustorium contact with the host xylem, Scale bars, 300 μm.
