# Supplementary figures and images for: Interspecific Signaling Between the Parasitic Plant and the Host Plants Regulate Xylem Vessel Cell Differentiation in Haustoria of Cuscuta campestris
Source: Front Plant Sci. 2020 Mar 13;11:193. doi: 10.3389/fpls.2020.00193 (PMC7082356; doi:10.3389/fpls.2020.00193)

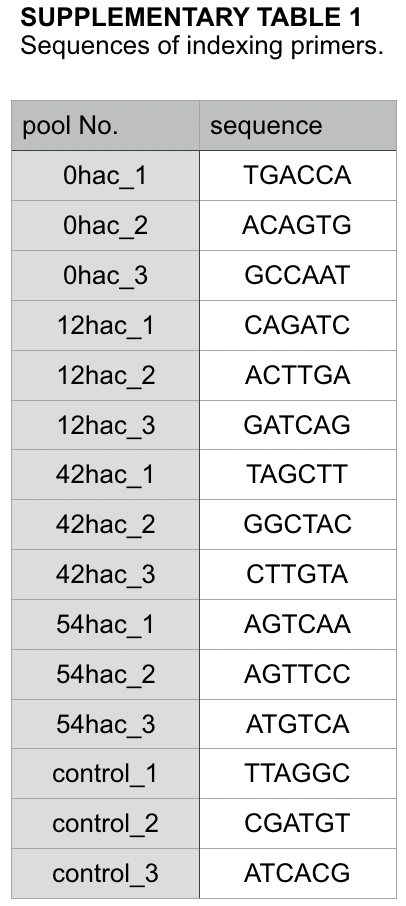

Supplement: Supplementary Table 1 — Sequences of indexing primers. [file Image_4.tiff]

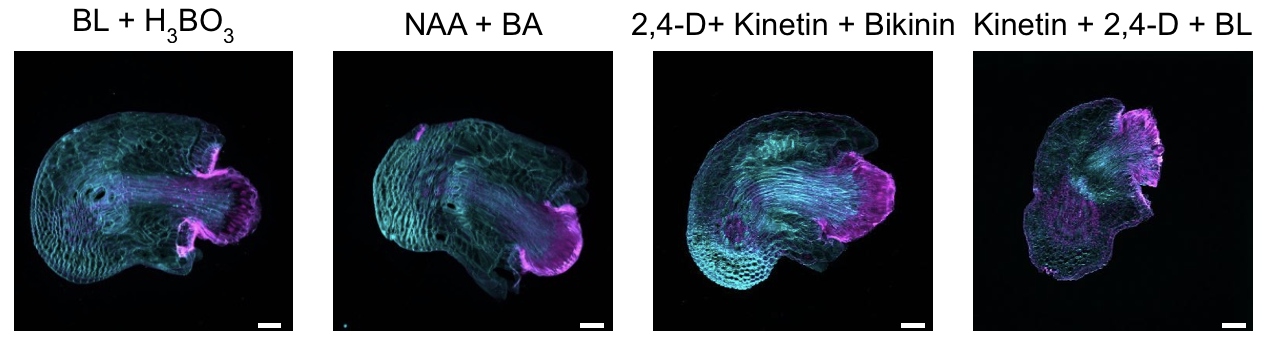

Supplement: Supplementary Figure 1 — Haustoria produced by the in vitro induction system were further incubated on a solid agarose medium containing phytohormones/chemicals mixtures for 48 hours. Digital accumulation images of Z-serial optical sections of the haustorium. Scale bars; 100 μm. BL, brassinolide; NAA, naphthaleneacetic acid; BA, benzyladenine; 2,4D, 2,4-dichlorophenoxyacetic acid. [file Image_1.tiff]

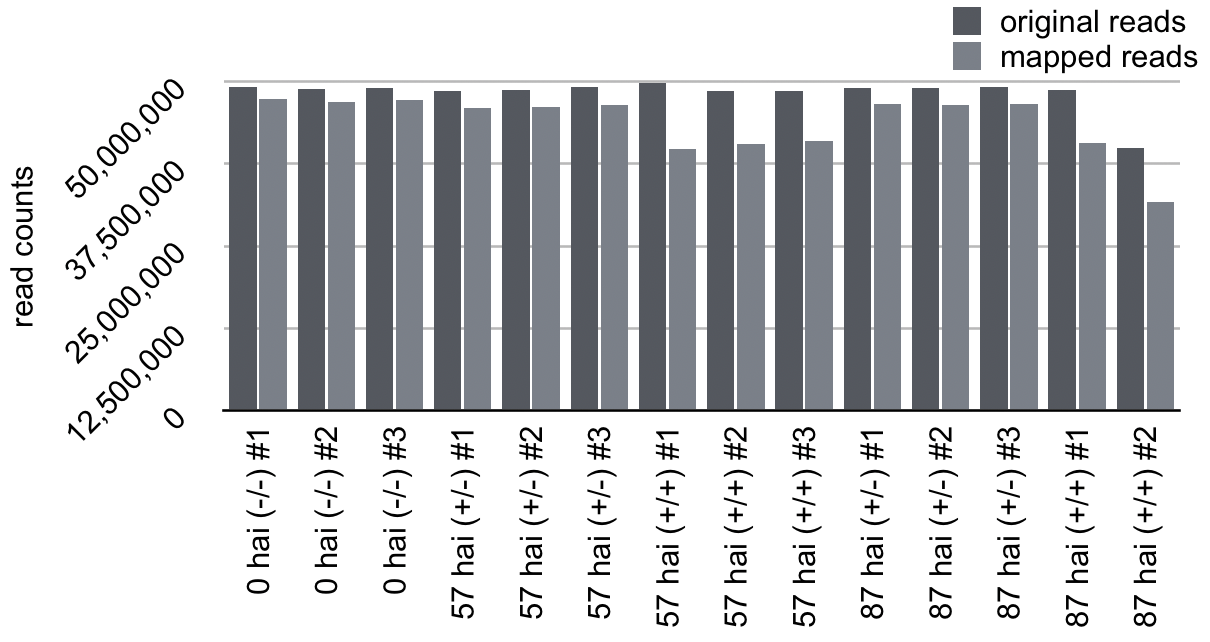

Supplement: Supplementary Figure 2 — Mapping counts of reads for each library against the C. campestris genome. [file Image_2.tiff]

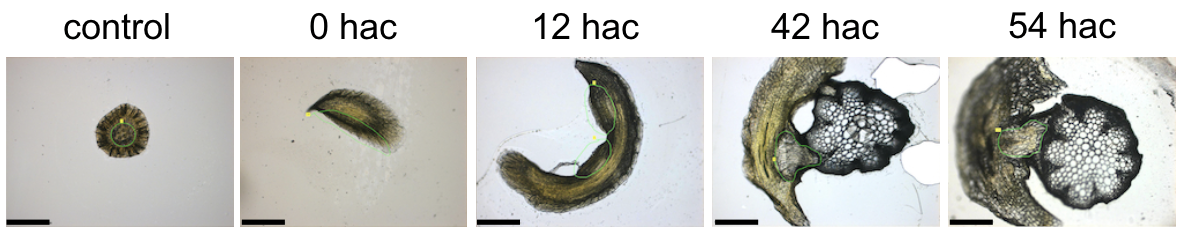

Supplement: Supplementary Figure 3 — Tissue samples from haustorium development in an intact host for RNA-sequencing libraries. Samples for each stage of the haustorium development were collected from transverse sections of 100 μm thickness using laser microdissection. Outside of the green circle showed the collected region for the control sample. Inside of the green circles showed the collected regions at 0, 12, 42, and 54 hours after coiling (hac). control, epidermal and cortical cells of C. campestris shoot irradiated with blue light for 24 hours; 0 hac, epidermal and cortical cells of the contact site with the host inflorescence stem just after the coiling; 12 hac, prehaustorium; 42 hac, haustorium just after penetration into the host; 54 hac, haustorium contact with the host xylem, Scale bars, 300 μm. [file Image_3.tiff]
